# Fetal bovine serum albumin inhibits antimicrobial peptide activity and binds drug only in complex with α1-antitrypsin

**DOI:** 10.1038/s41598-020-80540-6

**Published:** 2021-01-14

**Authors:** Wen-Hung Tang, Chiu-Feng Wang, You-Di Liao

**Affiliations:** Institute of Biomedical Sciences, Academia Sinica, Taipei, 115 Taiwan

**Keywords:** Biochemistry, Microbiology, Medical research, Molecular medicine

## Abstract

Several antimicrobial peptides (AMPs) have been developed for the treatment of infections caused by antibiotic-resistant microbes, but their applications are primarily limited to topical infections because in circulation they are bound and inhibited by serum proteins. Here we have found that some AMPs, such as TP4 from fish tilapia, and drugs, such as antipyretic ibuprofen, were bound by bovine serum albumin only in complex with α1-antitrypsin which is linked by disulfide bond. They existed in dimeric complex (2 albumin -2 α1-antitrypsin) in the bovine serum only at fetal stage, but not after birth. The hydrophobic residues of TP4 were responsible for its binding to the complex. Since bovine serum is a major supplement in most cell culture media, therefore the existence and depletion of active albumin/α1-antitrypsin complex are very important for the assay and production of biomolecules.

## Introduction

The widespread use of antibiotics in both medicine and agriculture has contributed to the emergence of drug-resistant bacteria^1,2^. Thus, development of new antimicrobials with unique targets and mechanism of action that are different from those of conventional antibiotics is urgently needed. Natural antimicrobial peptides (AMPs) have been isolated from multiple sources. They possess amphipathic structure (cationic and hydrophobic) and consist of 10–50 amino acid residues in length. They are able to disrupt the membrane integrity of bacteria in few minutes even for antibiotic-resistant bacteria and not prone to inducing drug-resistance^2–4^. Several AMPs are currently under examination in phase III clinical trials for their effectiveness in treating microbial infections, such as Omiganan, Pexiganan and Iseganan. However, their applications are limited primarily to topical infections probably due to the inhibition by serum proteins^5–7^.

Albumin is the most abundant protein in serum (~ 45 mg/ml), and involved in the transportation of fatty acids, cholesterols, toxins and drugs in the blood stream. The human serum albumin consists of a single polypeptide chain with 585 amino acid residues, which is organized in three α–helical domains, I, II, III, each consisting of two subdomains A and B^8–10^. The amino acid sequences of human, bovine and mouse albumins are highly homologous with 70 to 76% identities. There are two drug-binding sites and seven fatty acids-binding sites on it. More than 90% of anticoagulation drug warfarin and antipyretic drug ibuprofen were bound to site I and site II of human albumin, respectively. The lipopeptide antibiotic, daptomycin, and some short AMPs are dominantly bound and inhibited by human serum albumin (90 to 94%) at site II ^11,12^.

In this study, we found that bovine albumin acts on some AMPs and antipyretic drugs ibuprofen only in complex with α1-antitrypsin but not in free form. The complex exists in the bovine serum only at fetal stage but not after birth. Most importantly, the fetal bovine serum (FBS) is the major supplement of cell culture media, thus the content of albumin/α1-antitrypsin complex is very important for the assay and production of important biomolecules.

## Results

### Inhibition of bactericidal activities of AMPs by serum

**Figure 1:**
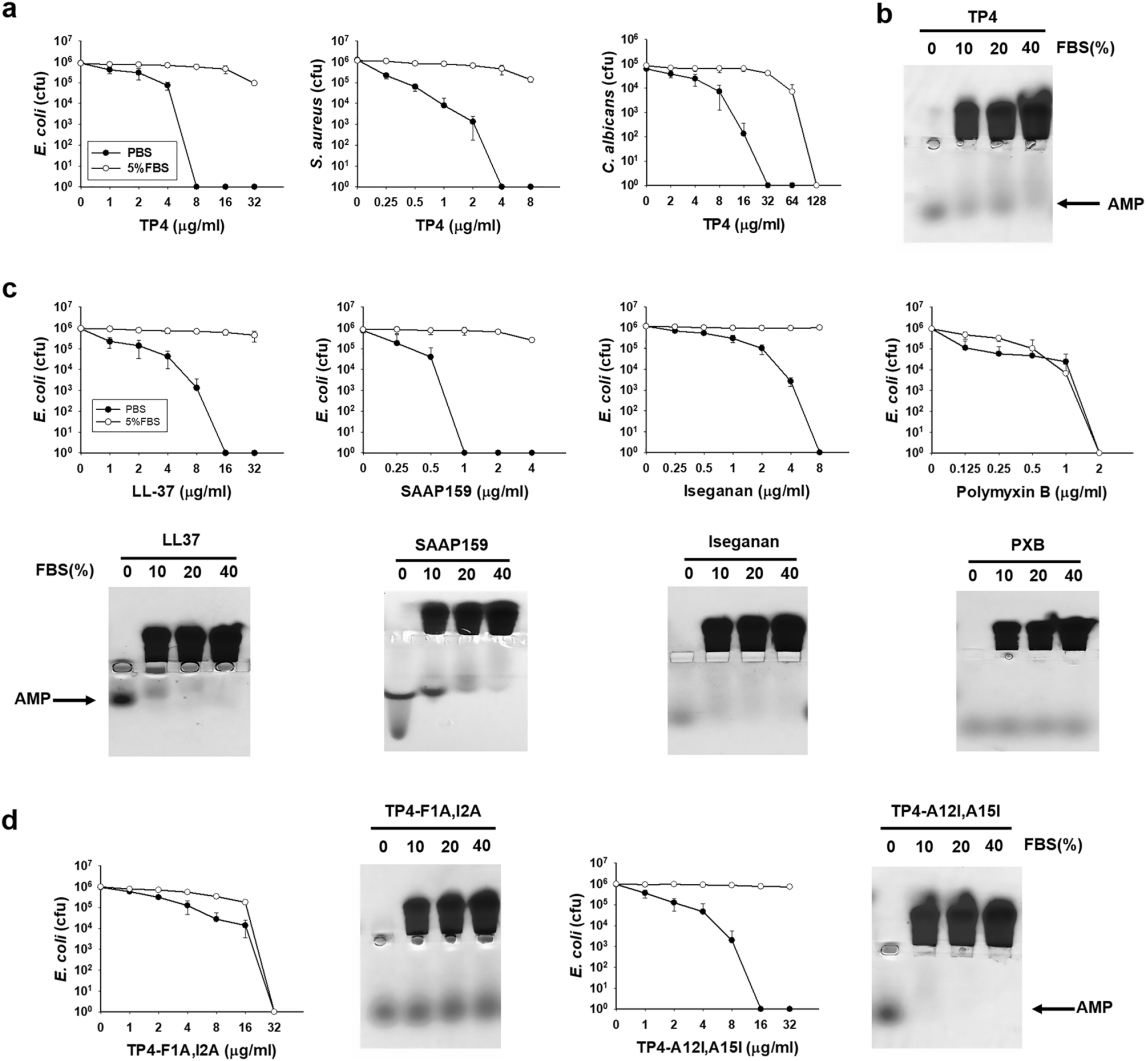
Susceptibility of antimicrobials to serum inhibition. (**a**) Effect of fetal bovine serum on the antimicrobial activity of TP4 to *E. coli* (left), *Staphylococcus aureus* (middle) bacteria and *Candida albicans* (right). (**b**) The band shift of TP4 caused by fetal bovine serum. (**c**, **d**) Effect of fetal bovine serum on the antimicrobial activities of cationic antimicrobial peptides and TP4 mutants to *E. coli* (top or left panel) and band shift of antimicrobials (4 μg each) caused by serum (bottom or right panel). PBS, phosphate-buffered saline; 5% FBS, 5% (v/v) fetal bovine serum in PBS.

The antimicrobial peptide (AMP) TP4 from fish tilapia exerts broad spectrum of antimicrobial activities against *E. coli, Staphylococcus aureus* and *Candida albicans.* However, these antimicrobial activities were markedly inhibited in the presence of fetal bovine serum (FBS) (5%, v/v) (Fig. 1a). In addition to TP4, this serum-mediated repression of AMPs against *E. coli* was also found with human AMP LL37, LL37-derived AMP (SAAP159) and protegrin-1-derived AMP (Iseganan), but not cyclic polypeptide antibiotic, Polymyxin B (Fig. 1c top panels). Using band shift assay by horizontal native gel electrophoresis, the mobility of LL37, SAAP159, Iseganan as well as TP4 was retarded in 10–40% FBS, however, that of Polymyxin B was not (Fig. 1b, c bottom panels). To investigate amino acid residues of TP4 that may be responsible for its susceptibility to serum’s inhibitory effects, specific hydrophobic and positive-charged residues of TP4 peptide were introduced or depleted and subjected to band shift assays. The results showed that deletion of hydrophobic residues, TP4-F1A,I2A, TP4-I5A,I6A and TP4-F9A,L10A, reduced the FBS-mediated shift, while the introduction of hydrophobic residue, TP4-A12I,A15I, enhanced the shift. In contrast, the deletion of cationic residue, TP4-R18S,R21H and TP4-dC2 (R24 and R25 deletion), did not change the AMP mobility (Fig. 1d and Supplementary Fig. 1). With respect to the bactericidal activity, the TP4-A12I,A15I became more susceptible to serum’s inhibition, while TP4-F1A,I2A was less susceptible (Fig. 1d). This result indicates that the hydrophobic residues of AMPs are responsible for the binding and inhibition caused by serum components.

### Purification of serum components responsible for AMP-binding

**Figure 2:**
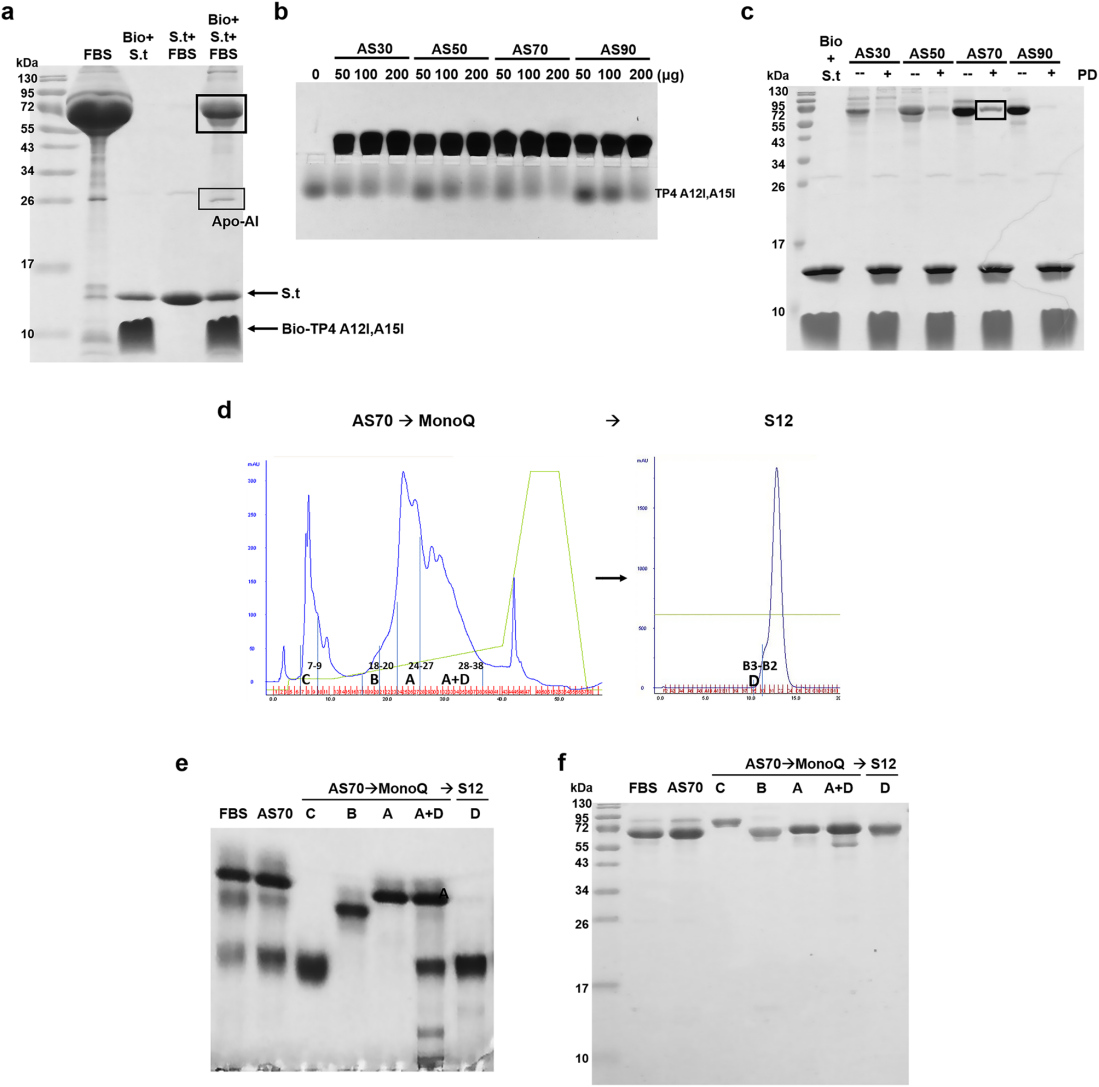
Purification of serum components responsible for AMP-binding. (**a**) Proteins bound to TP4-A12I,A15I peptides. The components in crude bovine serum were pulled down by biotinylated TP4-A12I,A15I peptides which were immobilized on Streptavidin-conjugated beads, and analyzed by 15% reduced SDS-PAGE. (**b**) Band shift of TP4-A12I,A15I peptides by serum proteins after ammonium sulfate fractionation. (**c**) Pulling down of serum proteins after ammonium sulfate fractionation by biotinylated TP4-A12I,A15I peptides which were immobilized on Streptavidin-conjugated beads. (**d**) FPLC chromatography for the purification of serum proteins from 70% saturated ammonium sulfate fractionation. Interested proteins (A, B, C and D) are shown on the profiles of FPLC Mono-Q (left) and S12 (right) column chromatography. (**e**, **f**) Homogeneity of purified serum components analyzed by horizontal 8% native PAGE, pH8.8, and vertical 15% reduced SDS-PAGE, respectively. Bio, biotinylated TP4-A12I,A15I; S.t., Streptavidin-conjugated beads; PD, pulling down; AS70, fractionation by 70% ammonia sulfate saturation.

Two bands, one major and one minor, were pulled down from the crude serum by biotinylated TP4-A12I,A15I peptides which had been immobilized on Streptavidin-conjugated beads (Fig. 2a). The major band consisted of albumin and α1-antitrypsin as determined by liquid chromatography-tandem mass spectrometry (LC/MS/MS), and the minor band was identified to be apolipoprotein AI (Apo-AI) (Supplementary Table 1). The active components responsible for TP4-A12I,A15I-binding were mainly located at the 70% ammonia sulfate saturation fraction as analyzed by band shift assay and pulling down experiment (Fig. 2b, c). The active components were further purified by fast performance liquid chromatography (FPLC) Mono Q and S12 column chromatography from the 70% saturated fraction (Fig. 2d). The active fractions of both column eluates (Supplementary Fig. 2a, e) were collected based on band shift assay (Supplementary Fig. 2d, h), such as fractions 32–40 of Mono Q and fractions 2–5 of S12 column chromatography. The protein components of each column eluate were analyzed by horizontal native PAGE (Supplementary Fig. 2b, f) and reduced SDS-PAGE (Supplementary Fig. 2c, g). The homogeneities of collected protein A (albumin), protein B (α1-antitrypsin), protein C (serotransferrin) and protein D complex (albumin and α1-antitrypsin) as shown on the elution profile of FPLC Mono Q column chromatography (Fig. 2d) were further analyzed by horizontal 8% native PAGE (Fig. 2e) and vertical 15% reduced SDS-PAGE (Fig. 2f). Detailed information for the validations of these proteins A, B, C and D is shown in Supplementary Table 1.

### AMP-binding and antitrypsin activities of proteins A, B and D

**Figure 3:**
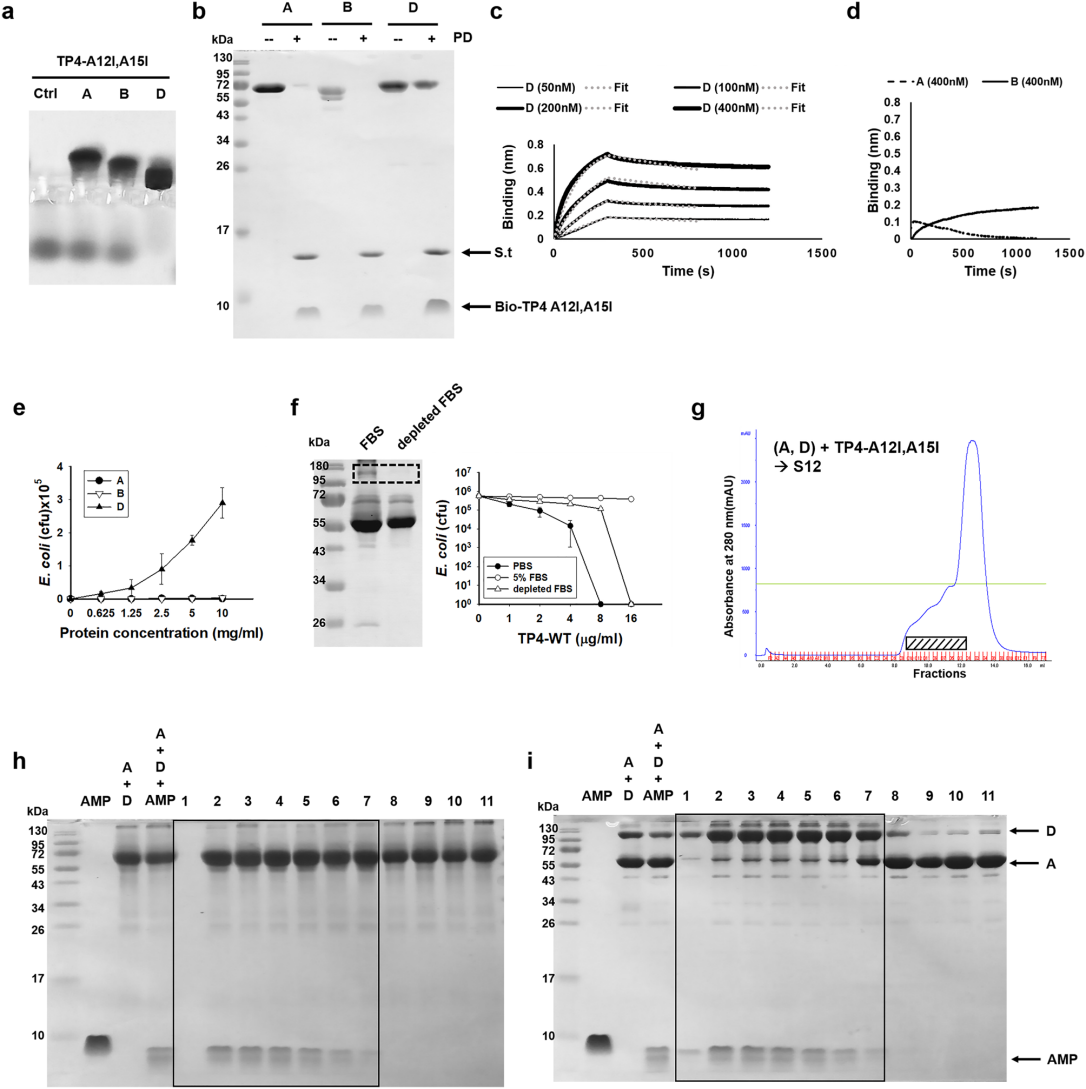
Binding of purified proteins to TP4-A12I,A15I. (**a**) Band shift of TP4-A12I,A15I by proteins A, B and D analyzed by 8% horizontal native PAGE and Coomassie blue staining. (**b**) Pulling down of proteins A, B and D by biotinylated TP4-TP4A12I,A15I immobilized on Streptavidin-conjugated beads. (**c**, **d**) Biolayer interferometry analysis of protein A, B, and D. The protein D showed potent and reversible binding to biotinylated TP4-A12I,A15I (**c**), but no substantial binding was observed in protein A and B (**d**). Heavy lines represent the experimental data, dotted lines represent the global-fitted data for analysis. (**e**) Inhibition of bactericidal activity of TP4 to *E. coli* (10^6^ cfu/ml) by protein D not by protein A or B. (**f**) Decrease of inhibitory effect of FBS on TP4′s antimicrobial activity by the depletion of protein D with TP4-A12I,A15I immobilized beads. (**g**) FPLC S12 gel filtration chromatography of proteins A/D mixture in the presence of TP4-A12I,A15I. (**h**, **i**) Analysis of column eluates as shown in figure **g** by 15% reduced (**h**) and non-reducing (**i**) SDS-PAGE. TP4-A12I,A15I-containing fractions are shown in box.

As shown, TP4-A12I,A15I was bound by protein D (albumin and α1-antitrypsin complex) only, but not by protein A (albumin) nor protein B (α1-antitrypsin) alone as determined by band shift and pulling down assays (Fig. 3a, b). It is of note that the binding ability of wild type TP4 to protein D was much less than that of TP4-A12I,A15I (Supplementary Fig. 3a, b). Similarly, the LL37, SAAP159 as well as Iseganan was also bound by protein D, but not by protein A and protein B. Furthermore, the introduction of hydrophobic residue, TP4-A12I,A15I, increased the binding ability to protein D, deletion of hydrophobic residues, TP4-F1A,I2A, TP4-I5A,I6A and TP4-F9A,L10A, reduced the binding ability. In contrast, the deletion of cationic residue, TP4-R18S,R21H and TP4-dC2, did not change the binding ability (Supplementary Fig. 3b). For the quantitation of binding affinity, the binding constant of protein D to immobilized biotinylated TP4-A12I,A15I was determined by biolayer interferometry with Kd = 21.7 nM, while that for protein A and B was not detectable (Fig. 3c, d). The bactericidal activity of TP4 (16 μg/ml) against *E. coli* (10^6^ cfu/ml) was inhibited by increasing concentrations of protein D, but not by protein A or B even up to 10 mg/ml (Fig. 3e). The bactericidal activity of TP4 was inhibited by 5% FBS, but the inhibition markedly decreased if the protein D as well as Apo-AI in the FBS was depleted by TP4-A12I,A15I-immobilized beads (Fig. 3f).

To further investigate the differential binding of AMP to protein A and protein D, the TP4-A12I,A15I and protein A/D was mixed and subjected to gel filtration chromatography (FPLC S12). There was a front shoulder on the elution profile (Fig. 3g). The albumin showed a broad distribution in the eluates, while the TP4-A12I,A15I co-eluted with albumin only at the front shoulder fractions analyzed by reduced SDS-PAGE (Fig. 3h). Interestingly, the albumin co-eluted with TP4-A12I,A15I (fractions 2–7) exhibited very slow mobility on the non-reducing SDS-PAGE with molecular mass close to 130kD which is much higher than that (< 72kD) after reduction with 2-mercaptoethanol (β-ME) (Fig. 3i). This result indicates that the TP4-associated albumin may accompany with other protein(s), such as α1-antitrypsin, through disulfide bond.

To see whether the protein B (α1-antitrypsin) in the protein D complex still possess antitrypsin activity after being bound by protein A (albumin), we found that the proteolytic activity of trypsin toward TP4 and a larger AMP SAAP159 was repressed by protein D as well as free protein B, but only minimally affected by protein A (albumin) as analyzed by 15% reduced SDS-PAGE (Supplementary Fig. 4).

**Figure 4:**
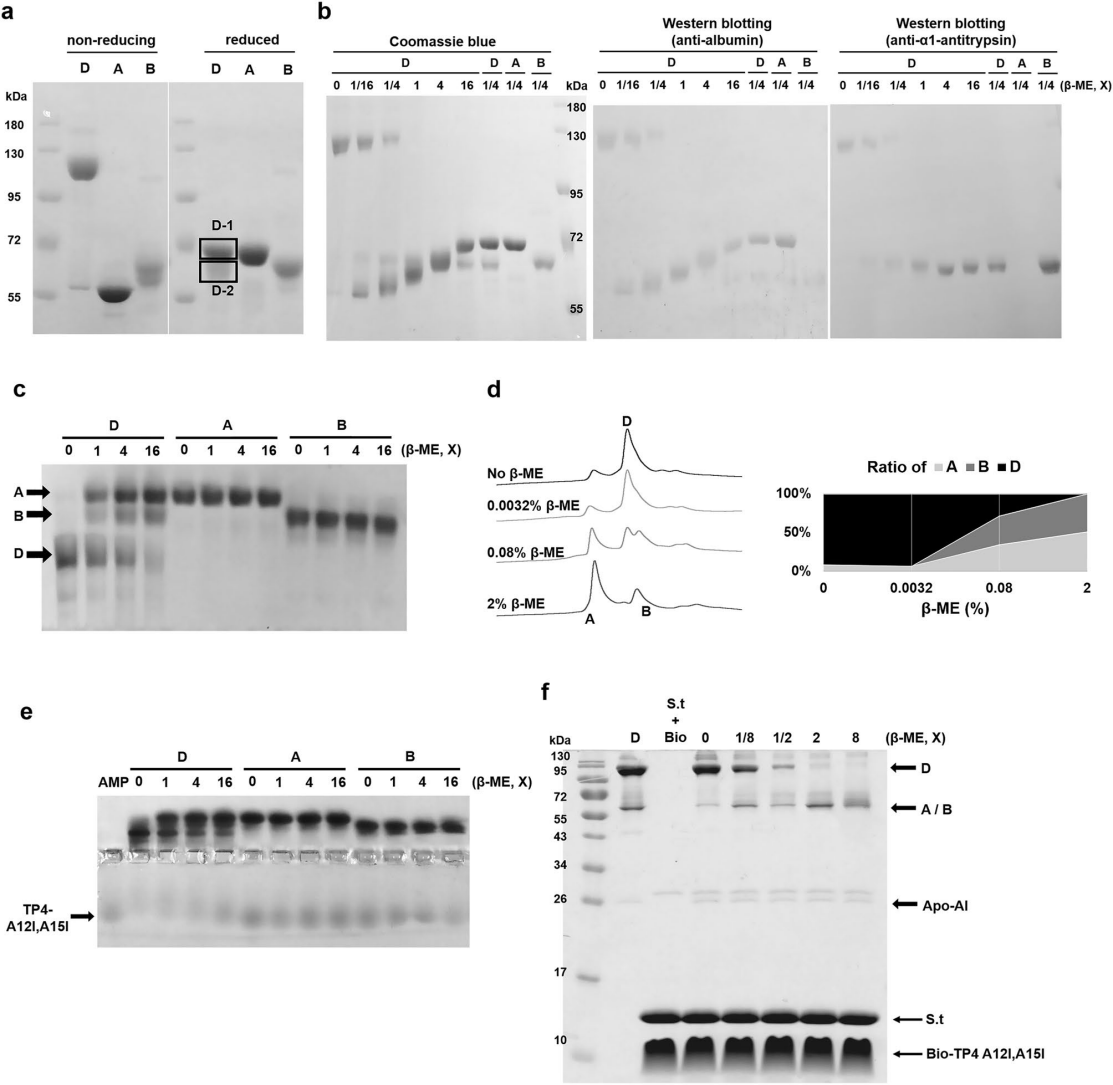
Protein D is composed of albumin and α1-antitrypsin linked by disulfide bond. (**a**) Protein D is dissociated into albumin and α1-antitrypsin by β-mercaptoethanol (β-ME). Proteins A, B and D (4 μg each) were analyzed by reduced (2.5% β-ME) (left) and non-reducing (right) 8% SDS-PAGE followed Coomassie blue staining. (**b**) Dissociation of protein D into protein A and B by increasing concentration of β-ME and analyzed by non-reducing SDS-PAGE (left) and Western blotting using antibodies raised against albumin (middle) and α1-antitrypsin (right). 1X represents 0.08% β-ME (v/v). (**c**) Dissociation of protein D into protein A and B by increasing concentration of β-ME and analyzed by 8% native PAGE, pH8.8. 1X represents 0.05% β-ME (v/v). (**d**) Separation of protein A and B from protein D by HPLC in the presence of β-ME. The elution profile of these dissociated proteins by HPLC (left) and the quantification of individual protein (right) were shown. (**e**, **f**) Decrease of AMP-binding ability of protein D by β-ME. The binding ability of proteins D, A and B to TP4-A12I,A15I peptide were determined by band shift and pulling down assays after reduction with β-ME. 1X represents 0.05% β-ME. Bio, biotinylated TP4-A12I,A15I.

### Disulfide linkage between albumin and α1-antitrypsin is essential for AMP-binding

Protein D exhibited one major band which moved very slowly and one minor band which moved faster than 72kD on the 8% non-reducing SDS-PAGE (Fig. 4a, left panel). However, it was separated into two bands (D1, D2) after reduction with β-ME and the two bands (D1 and D2) were identified to be albumin and α1-antitrypsin, respectively, by LC/MS/MS analysis (Fig. 4a, right panel and Supplementary Table 1). In addition, protein A (albumin) in non-reducing form moved faster than that in reduced form. To investigate the dynamic of protein D dissociation, increasing concentrations of β-ME were added to protein D and the resulting mixtures were subjected to non-reducing SDS-PAGE and Western blotting analyses using antibodies raised against albumin and α1-antitrypsin, respectively (Fig. 4b). The results demonstrate that protein D was dissociated into protein A and protein B by β-ME. Similarly, the dissociation of protein D by β-ME (0.05–0.8%) was also observed by horizontal native PAGE and high-performance liquid chromatography (HPLC-C4 column) (Fig. 4c, d). To investigate the role of disulfide bond, we found that AMP-binding ability of protein D was destroyed if it was reduced by β-ME as analyzed by band shift (Fig. 4e) and pulling down assay (Fig. 4f). It is of note that some protein D constituents were able to bind the TP4-A12I,A15I after being dissociated by β-ME (0.1–0.4%) (Fig. 4f). These results demonstrate that the disulfide bond between protein A (albumin) and protein B (α1-antitrypsin) is essential for the structural integrity and AMP-binding ability.

### Dimerization of protein D is essential for AMP-binding

**Figure 5:**
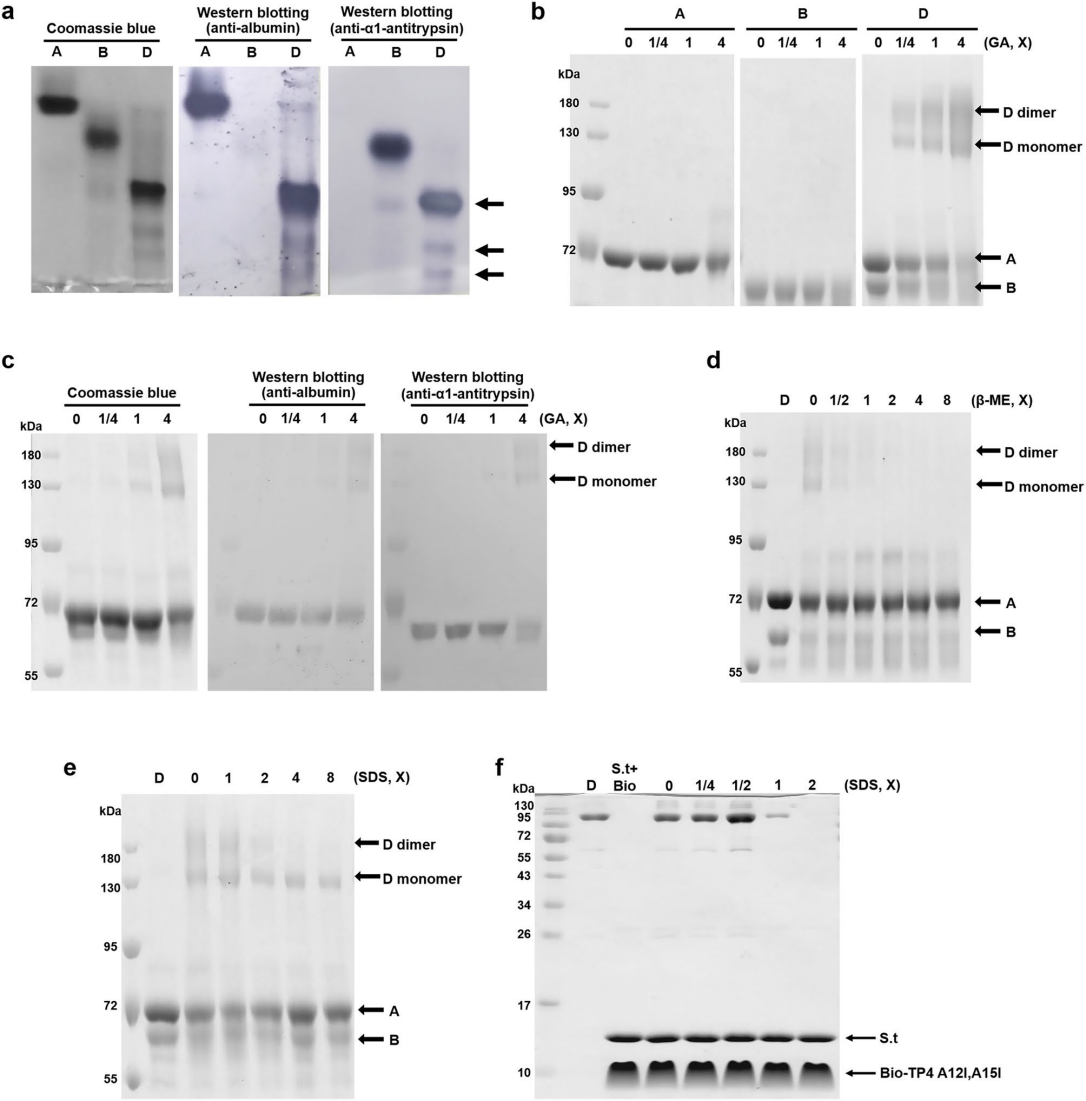
Dimerization of protein D is essential for AMP binding. (**a**) Protein D is composed of protein A and B detected by non-reducing SDS-PAGE followed by Western blotting using antibodies raised against protein A (albumin, middle) and protein B (α1-antitrypsin, right). (**b**, **c**) Dimerization of protein D. The proteins A, B and D were cross-linked with glutaraldehyde (GA) and separated by 8% reduced SDS-PAGE (**b**). The cross-linked protein D was further analyzed by Western blotting using antibodies raised against protein A (albumin, middle) and protein B (α1-antitrypsin, right) (**c**). 1X GA represents 0.00625% glutaraldehyde. (**d**, **e**) Inhibition of dimerization of protein D by β-mercaptoethanol (β-ME) and SDS. The protein D was cross-linked with 0.025% glutaraldehyde in the presence of β-ME (**d**) or SDS (**e**). 1X represents 0.05% β-ME (**d**) and 0.0625% SDS (**e**), respectively. (**f**) SDS inhibit the pulled-down of protein D to immobilized TP4-A12I,A15I which analyzed by 15% non-reducing SDS-PAGE. 1X represents 0.0625% SDS.

The non-reduced protein D was recognized by both antibodies raised against albumin and α1-antitrypsin. One major band as well as two minor bands were observed (Fig. 5a). The protein D (A + B complex) existed in monomeric and dimeric forms as shown on the reduced SDS-PAGE after being cross-linked with glutaraldehyde. In contrast, proteins A and B still existed as monomers even after being cross-linked as was done with protein D (Fig. 5b). The monomeric and dimeric forms of protein D were further validated by Western blotting using respective antibodies raised against protein A (albumin) or protein B (α1-antitrypsin) (Fig. 5c). The dimerization of protein D was inhibited by 2 × SDS, equivalent to 0.125% SDS (w/v), as well as 0.05% β-ME (w/v) where protein D was dissociated into protein A and B (Fig. 5d, e). Under the environments (2 × SDS) in which dimerization was inhibited, protein D was unable to bind to TP4-A12I,A15I as analyzed by pulling down assay (Fig. 5f). These results indicate that dimerization of protein D is essential for its AMP-binding activity.

### TP4 peptide binding sites on protein D

**Figure 6:**
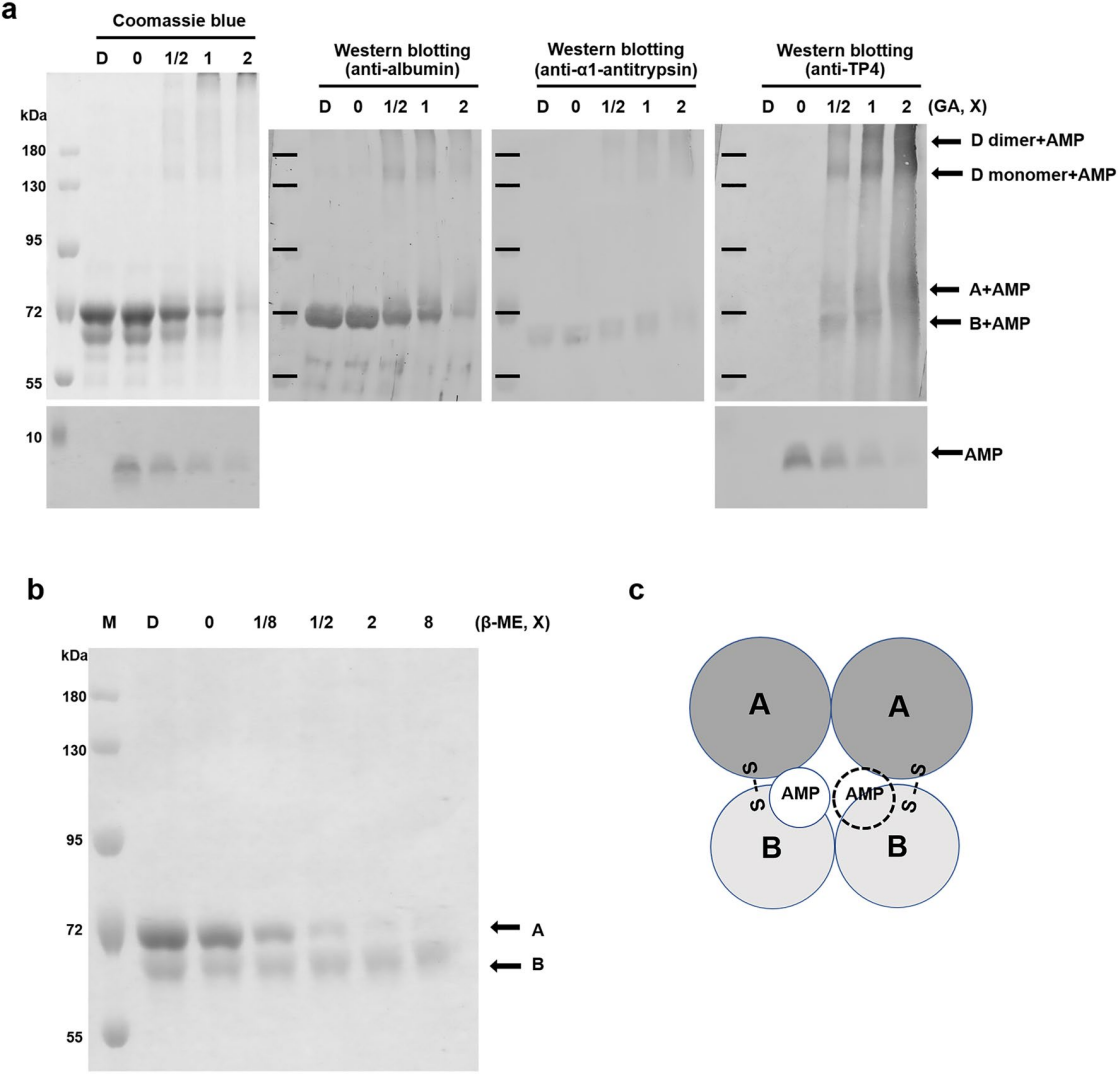
Binding of TP4-A12I,A15I peptides to the albumin/α1-antitrypsin complex. (**a**) Binding of TP4-A12I,A15I peptides to protein D. The AMP-protein D complexes were cross-linked with glutaraldehyde (GA), separated by 8% or 15% reduced SDS-PAGE, stained by Coomassie blue and detected by Western blotting using antibodies raised against albumin, α1-antitrypsin and TP4, respectively. 1X represents 0.00625% GA. (**b**) Binding of TP4-A12I,A15I peptides to the α1-antitrypsin subunit. The protein D was pulled down by immobilized biotinylated TP4-A12I,A15I on Streptavidin beads. The retained proteins after β-mercaptoethanol (β-ME) wash were analyzed by 8% reduced SDS-PAGE and Coomassie blue staining. 1X represents 0.25% β-ME. (**c**) Model for the interaction between AMP and protein D. The active complex is composed of two identical protein D and each subunit is composed of protein A (albumin) and protein B (α1-antitrypsin) linked by disulfide bond.

To elucidate the interactions between TP4-A12I,A15I and protein D (albumin and α1-antitrypsin), the AMP/protein D complex was cross-linked with glutaraldehyde, separated by reduced SDS-PAGE and analyzed by Western blotting using antibodies raised against albumin, α1-antitrypsin and TP4 (Fig. 6a). Interestingly, TP4-A12I,A15I was associated with protein A, protein B as well as monomeric and dimeric protein D (Fig. 6a). However, only protein B was still tightly associated with the immobilized TP4-A12I,A15I if the pulled down-protein D was dissociated by β-ME (Fig. 6b). These results suggest that TP4-A12I,A15I is close to protein B in the albumin and α1-antitrypsin complex (Fig. 6c).

### Binding site of AMP and drug in protein D

**Figure 7:**
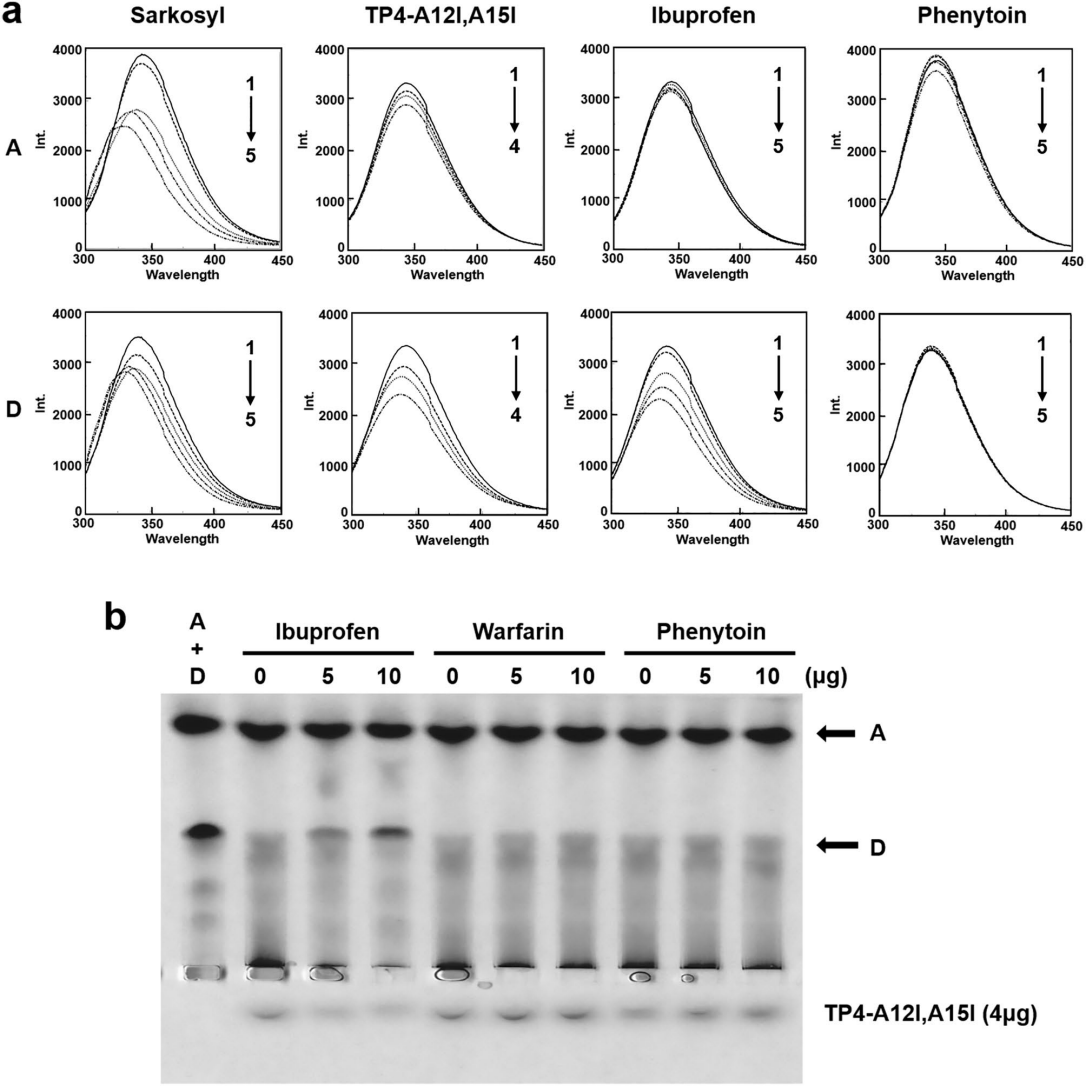
Binding of TP4-A12I,A15I peptides and drug to protein D. (**a**) Emission spectra of proteins A and D in the presence of sarkosyl, TP4-A12I,A15I, Ibuprofen and Phenytoin. Reagents were added to the protein A and protein D at the indicated concentrations and excited at 280 nm and recorded for the emission spectrum between 300 to 450 nm. Protein A and D: 0.75 μM each; Sarkosyl: lines 1–5 at 0, 5, 20, 80 and 320 μM; TP4-A12I, A15I: lines 1 to 4 at 0, 30, 60, 120 μM; Ibuprofen; lines 1 to 5 at 0, 3, 6, 12 and 24 μM; Phenytoin; lines 1 to 5 at 0, 2.5, 5, 10 and 20 μM. (**b**) Inhibition of the binding of TP4-A12I,A15I to protein D by Ibuprofen. The protein A and D mixture (20 μg each) was pre-incubated with Ibuprofen, Warfarin or Phenytoin for 30 min before incubating with TP4-A12I,A15I (4 μg) at 37 °C for 30 min, and analyzed by native PAGE, pH8.8.

The blue shift of fluorescence emission spectrum of protein D was observed following the addition of TP4-A12I,A15I or antipyretic agent ibuprofen, but these reagents did not cause blue shift in protein A (albumin)^13^. As a positive control, the anionic surfactant, sarkosyl, caused blue shifts for both protein A and protein D (Fig. 7a). In contrast, no blue shift was seen in protein A and protein D by the anticonvulsant phenytoin which is weekly bound by albumin^14^**.** Furthermore, only protein D, but not protein A, was bound by TP4-A12I,A15I analyzed by band-shifted assay. Interestingly, the binding of protein D by TP4-A12I,A15I was competitively inhibited by ibuprofen, but not by warfarin or phenytoin (Fig. 7b). These results suggest that TP4-A12I,A15I and ibuprofen may share the same binding site probably at the drug-binding site II of albumin in the protein D complex.

### Variation of protein D content in bovine serum

**Figure 8:**
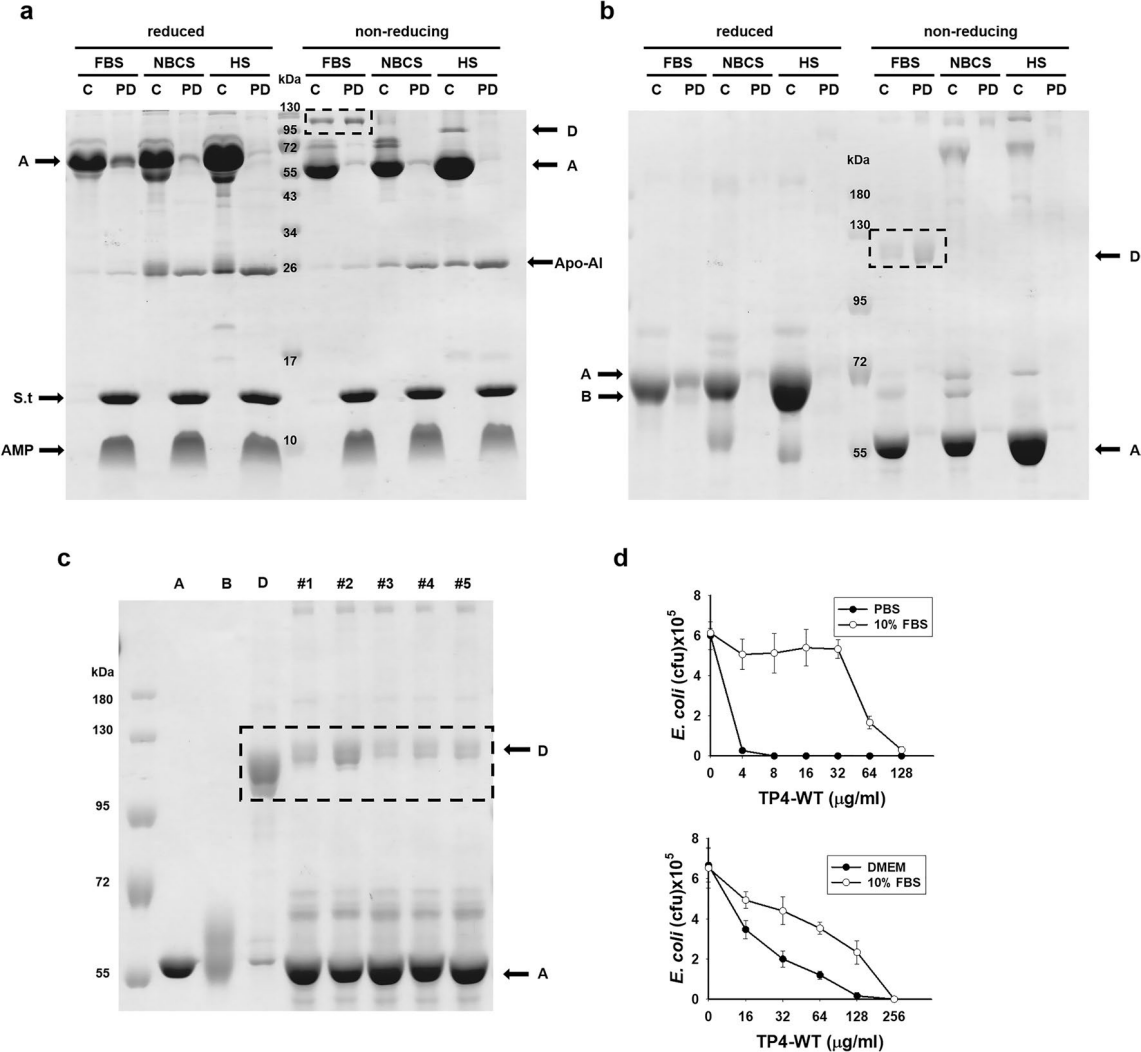
Analyses of albumin and α1–antitrypsin complex in the sera. (**a**, **b**) 0.25 μl of fetal bovine serum (FBS), newborn calf serum (NBCS) and human serum (HS) were pulled down by biotinylated TP4-A12I,A15I immobilized on Streptavidin-conjugated gel and subjected to 15% (**a**) and 8% (**b**) reduced (2.5% β-ME) and non-reducing SDS-PAGE analyses. C, control serum without pulling down; PD, pulling down (**c**) Analysis of the protein A and D contents of various FBS. 0.25 μl of crude FBS was analyzed by 8% non-reducing SDS-PAGE. # 1 to # 5 were obtained from Gibco, Co. with the lot numbers 1233075 and 1,887,129, CORNIING with the lot number 35010170, Biological Industries with the lot numbers 1823627 and 1,650,071, respectively. (**d**) Susceptibility of TP4 to serum inhibition in PBS and cell culture medium (DMEM).

The bovine serum is the major supplement of cell culture media which is generally used in modern medical researches for the assay and production of biomolecules. We are interested in the amount of protein D in the bovine serum at different developmental stages or sources of vender. The protein D was seen only in the bovine serum at the fetal stage but not after birth analyzed by non-reducing 15% and 8% SDS-PAGE and pulling down experiments (Fig. 8a, b). The homologous protein D was not seen in the serum of adult human. Interestingly, the Apo-AI was minimally seen in FBS, but it markedly increased in calf serum as that in human serum (Fig. 8a). Furthermore, the ratio of protein D to A in FBS varied with serum batch or source of vender (Gibco, CORNIING and Biological industries) as analyzed by non-reducing SDS-PAGE (Fig. 8c). It is suggested that the AMP- or drug-binding capacity of serum may vary with the source of serum.

In PBS, the antimicrobial activity of TP4 against *E. coli* was markedly repressed by 10% FBS with 16-folds increase in MBC. Interestingly, the TP4 activity was also markedly inhibited with 32-folds increase in MBC if the assay is performed in DMEM medium alone without FBS addition (Fig. 8d). Similarly, the bactericidal activity of TP4 was further repressed by the addition of 10% FBS in DMEM which is generally used for cell culture (Fig. 8d).

## Discussion

Albumin is the most abundant protein in human serum (HSA, 45 mg/ml), followed in descending order by α1-antitrypsin (A1AT, 3.5 mg/ml), transferrin (2.5 mg/ml), α2-macroglobulin (2.5 mg/ml), haptoglobulin (1.8 mg/ml), low-density lipoprotein (LDL, 1.2 mg/ml), α1-acid glycoprotein (AGP, 0.8 mg/ml) and high-density lipoprotein (HDL, 0.6 mg/ml)^11^**.** These proteins are involved in the transportation of fatty acids, cholesterols, toxins and drugs in the blood stream. More than 90% of anticoagulation drug warfarin and antipyretic drug ibuprofen were shown to bind to site I and site II of human albumin, respectively^8–10^. However, the binding ability of anticonvulsant drug phenytoin to albumin is low although 90% of the drug is bound to serum protein^14^. The lipopeptide antibiotics daptomycin is highly bound to human serum components (90 to 94%)^15^ in the following order, albumin >  > α1-antitrypsin > low-density lipoprotein > high-density lipoprotein > transferrin^11^. In addition, some antibiotics, such as ceftriaxone and moxifloxacin, and short cationic AMPs, such as CAP1-CAP9, are also bound and inhibited by human and bovine serum albumin^12,16,17^. In this study we found that the fetal bovine serum albumin was active in the binding and inhibition of AMP activity only in complex with α1-antitrypsin, whereas the free form albumin or free α1-antitrypsin alone was almost unable to exert these binding properties when analyzed by all the following detection methods: band shift, pulling down, biolayer interferometry, gel filtration chromatography, bactericidal activity and blue shifts of fluorescence emission spectrum.

The existence of active albumin and α1-antitrypsin complex has not been reported in human or bovine serum yet. Our results also show that the relative amounts of free and complex albumin in the serum varied with the developmental stage of bovine, batches of serum sources (Fig. 8). Therefore, the amount of albumin/α1-antitrypsin complex is very important because bovine serum is a major supplement of culture media which is used for the assay and production of biomolecules. In addition to the protein D (albumin/α1-antitrypsin complex), the culture medium (DMEM) which contains salts and divalent cations is also inhibitory to the antimicrobial activity of TP4 (Fig. 8). To prevent the inhibitory effects of serum component on the antimicrobial activity of AMP, the protein D in the FBS may be depleted by the AMP-immobilized beads before adding to the culture media, DMEM or RPMI.

The band shift of TP4-A12I,A15I on native PAGE occurred with bovine protein D, but not with protein A (Fig. 3a). Interestingly, the binding between protein D and TP4-A12I,A15I was blocked if the protein D-containing mixture was pre-incubated with ibuprofen, but not with warfarin or phenytoin, before binding to TP4-A12I,A15I (Fig. 7b). It is known that warfarin and ibuprofen bind to human albumin at the binding site I and II, respectively^10^. Furthermore, the conformation changes of albumin could be monitored by the blue shift of fluorescence emission spectrum exerted by tryptophan residues. The movement of tryptophan residues, W131 and W214 located in sites I and II of BSA, induced by drug treatment could be detected by the assay^13,18^. For example, the anionic surfactant sarkosyl drove the blue shifts in both proteins A and D (Fig. 7a). The TP4-A12I,A15I and ibuprofen drove blue shift only in bovine protein D, but not protein A, whereas phenytoin did not exert blue shift in both proteins. These results suggest that the TP4-A12I,A15I and ibuprofen share the same binding site probably at the binding site II of albumin in protein D.

The α1-antitrypsin belongs to the serpin family being inhibitors of serine proteinases. Both protein B (free form α1-antitrypsin) and protein D (complex form) exerted anti-proteolytic activity (supplementary Fig. 4). It is a glycoprotein with 394 amino acid residues and composed of 3 β–sheets and 9 α-helices having only one cysteine at Cys232 for disulfide linkage with albumin which contains 17 pairs of disulfide bond and one free cysteine^19^. The TP4 signals were seen on the A and B subunits as well as on monomeric and dimeric forms of protein D as detected by Western blotting after being cross-linked by glutaraldehyde (Fig. 6a). However, protein B still bound to TP4-A12I,A15I if protein A was removed from the AMP-protein D complex by β-ME (Fig. 6b). It indicates that AMP lies close to protein B (α1-antitrypsin) in the complex. Furthermore, the dimerization of two identical protein D molecules was important for AMP-binding since the pulling down of protein D by immobilized AMP was inhibited by anionic surfactant SDS which disrupts hydrophobic interaction between these two subunits (Fig. 5e, f). The model of AMP binding in the complex is proposed as shown in Fig. 6c. The complex is composed of two identical protein D molecules tethered by hydrophobic interaction, each protein D subunit consisting of protein A and B linked by disulfide bond.

In addition to protein D, the serum protein apolipoprotein AI (Apo-AI) was also pulled down from crude fetal bovine serum by immobilized TP4-A12I,A15I, but in a less amount (Fig. 2a). The Apo-AI with Mr = 28,080 Da is the major constituent of high-density lipoprotein (HDL), which is involved in the removal of cholesterol from peripheral tissues and its catabolism. The protein has been shown to bind human AMP, LL37, in an 1:1 complex and inhibits its antimicrobial and cytotoxic activities^20,21^. The amount of Apo-A1 in fetal bovine serum was much less than that of protein D (Fig. 8a, b). Interestingly, the amount of protein D decreased and Apo-Al increased in the bovine serum after birth. It is of note that the absence of protein D and presence of Apo-Al were also seen in adult human serum (Fig. 8a). It is suggested that protein D may play important roles in the development of fetus in bovine and probably in human, while the Apo-AI in HDL may play roles in the neutralization and transportation of AMPs and drugs in the sera of bovine and human after birth.

In conclusion, the albumin is active in AMP-binding only in complex with α1-antitrypsin in bovine serum at fetal stage, but not after birth. It is composed of two identical albumin-α1-antitrypsin subunits linked by disulfide bond. The amount of albumin/α1-antitrypsin complex in bovine serum is very important and may be depleted by AMP-immobilized beads because FBS is a major supplement for cell culture media which is used for the assay and production of biomolecules.

## Methods

### Materials

Fetal bovine sera (FBS) were obtained from the following sources: Gibco (New York, USA) with lot numbers 1233075 and 1,887,129, CORNIING (New York, USA) with lot number 35010170, and Biological Industries (Israel) with lot numbers 1823627 and 1,650,071. Newborn calf serum was obtained from Life Biotechnologies Corporation (California, USA) with catalogue No. 16010167 and lot number 1999580. The rabbit antibodies raised against bovine serum albumin and human α1-antitrypsin were obtained from GeneTex (San Antonia, USA) with catalogue numbers GTX 79,816 and GTX 112,707, respectively. The rabbit antibodies raised against TP4 was prepared by Yao-Hong Biotechnology (New Taipei, Taiwan) using synthetic TP4 polypeptide. Streptavidin-conjugated beads, FPLC columns (Mono Q and Superose 12™) were obtained from GE Healthcare Bio-sciences AB (Uppsala, Sweden). Antimicrobial peptides, such as TP4^22^, LL37^23^, Iseganan^24^, and SAAP159^7^ were synthesized by Kelowna International Scientific Inc. (Taipei, Taiwan) with more than 95% purity and their molecular masses were verified by mass spectrum analysis. Human serum was collected and prepared from adult human blood using the protocol approved by Academia Sinica IRB (AS-IRB01-18,072) at 2020, Aug.21. Ibuprofen and Phenytoin was purchased from Tokyo Chemical Industry (Tokyo, Japan) with catalogue numbers I0415 and D0894, respectively. Polymyxin B (A2250) and Warfarin were purchased from Sigma-Aldrich (St. Louis, Missouri, USA).

### Antimicrobial activity assays

The Gram-negative bacteria *Escherichia coli K-12* (MG1655) was cultured and plated in/on Luria–Bertani broth/agar plate (Merck Millipore, Darmstadt, Germany). The Gram-positive *Staphylococcus aureus* (ATCC 6538P) was cultured and plated in tryptic soy broth/agar (BD, MD, USA). The fungus *Candida albicans* (ATCC 14,053) was cultured and plated in YM broth/agar (BD, MD, USA). The bacteria were grown overnight, washed, and diluted 1:2000 in phosphate buffered saline (PBS), pH 7.5 or DMEM (Life Biotech, California, USA), pH 7.4. Bacteria (ca. 1 × 10^6^ colony-forming units, cfu) were mixed with antimicrobial peptides (AMP) in 100 μl PBS and incubated at 37 °C (for bacteria) and at 30 °C (for fungus). Serial dilutions of each AMP-treated bacteria in PBS/DMEM supplemented with FBS were prepared and plated for the determination of remaining viable cells (expressed as cfu). At least three independent experiments were performed for each assay to determine the average value with standard deviation^25^. MBC was defined as the lowest AMP concentration that kills 99.9% of the original inoculum.

### Purification of serum proteins by ammonium sulfate fractionation and column chromatography

Ammonium sulfate were added to aliquots of FBS at 30%, 50%, 70% and 90% saturation and incubated on ice for 30 min with occasional agitation. The precipitates were collected by centrifugation at 12000xg, 4 °C for 10 min, and dialyzed against PC buffer (20 mM HEPES, 50 mM NaCl, pH7.4) and stored at -80 °C before use. The fraction of 70% ammonium sulfate-saturated components was further purified by fast protein liquid chromatography (FPLC) Mono Q and Superose 12™ (S12) column chromatographies.

### Binding shift assay

Various AMPs (4 μg each) were incubated with fetal bovine serum (FBS) components at room temperature in 10 μl PC buffer for 30 min and subjected to horizontal native 8% PAGE, pH8.0 or pH8.8, and Coomassie Blue staining.

### Identification of TP4-binding proteins in FBS

Streptavidin-conjugated beads were incubated with 10 μg of biotinylated TP4-A12I,A15I peptides in 600 μl PC buffer for one hour on a rolling wheel. The immobilized biotinylated AMPs were further mixed with serum components in the indicated 600 μl buffer(s) at room temperature for 30 min on a rolling wheel, then washed twice with 600 μl respective buffer and subjected to SDS-PAGE and Coomassie Blue staining. Specific proteins were excised from SDS-PAGE gel and subjected to in-gel trypsin digestion and liquid chromatography-tandem mass spectrometry^26^.

### Binding kinetics of protein to biotinylated TP4-A12I,A15I

The biolayer interferometry were measured by an Octet RED384 instrument (ForteBio). Biotinylated TP4-A12I,A15I (10 μM) were immobilized on a Streptavidin biosensor. Serial dilutions of serum components in 200 μl PC buffer were used as analyte. Affinity (Kd) and kinetic parameters (k_on_ and k_off_) were calculated from a global fit (1:1) of the data using the Octet RED384 software^27^.

### Antitrypsin activity analysis

The inhibitions of trypsin’s proteolytic activity by serum components on peptides TP4 and SAAP159 were determined as follows. Freshly diluted trypsin (2 ng; 0.2 μg/ml) was pre-incubated with various concentrations of serum components in PC buffer for 30 min before the mixture was added to TP4 (2 μg; 0.2 mg/ml) or SAAP159 (4 μg/; 0.4 mg/ml) peptides in 10 μl PC buffer at 37 °C for 30 min. The digested peptide fragments were analyzed by 15% reduced SDS-PAGE and Coomassie Blue staining.

### Cross-linking assay

Small aliquots of serum components (4 μg) in the presence/absence of AMPs (2 μg) were incubated with various concentrations of glutaraldehyde in 10 μl PC buffer at 37 °C for 30 min. The cross-linked complexes were analyzed by reduced SDS-PAGE and Coomassie Blue staining^28^.

### High-performance liquid chromatography

The 2-mercaptoethanol-treated samples in 50 μl was uploaded into a C4 column (250 mm × 4.6 mm, 5 μm, Vydac) at 37 °C, washed with A buffer (0.1% trifluoroacetic acid and 2% acetonitrile, v/v), eluted with a gradient of B buffer (0.1% trifluoroacetic acid and 98% acetonitrile, v/v) at a flow rate of 1 ml/min and detected at the wavelength of 220 nm. The relative amount of serum components was quantified by Agilent ChemStation program.

### Fluorescence emission spectrum measurement

The serum components (protein A or D, 0.75 μM each) were incubated with various concentrations of reagent, such as sarkosyl, TP4-A12I,A15I, ibuprofen and phenytoin, in 200 μl PC buffer at 25 °C for 30 min. The mixtures were excited by 285 nm and its emission spectra were recorded between 300 and 450 nm using a temperature-controlled fluorimeter (FP8500, Jasco, Japan)^13^.

### Data availability

The data that support the findings of this study are available from the corresponding author upon reasonable request.

## Supplementary Information

Supplementary files.
