# Supplementary material for: Fetal bovine serum albumin inhibits antimicrobial peptide activity and binds drug only in complex with α1-antitrypsin
Source: Sci Rep. 2021 Jan 14;11:1267. doi: 10.1038/s41598-020-80540-6 (PMC7809101; doi:10.1038/s41598-020-80540-6)
Supplement: Supplementary file 1 — Supplementary files. [file 41598_2020_80540_MOESM1_ESM.docx]

Supplementary material

**Fetal bovine serum albumin inhibits antimicrobial peptide activity and binds drug only in complex with α1-antitrypsin**

Wen-Hung Tang^1^, Chiu-Feng Wang^1^ and You-Di Liao^1*^

1. Institute of Biomedical Sciences, Academia Sinica, Taipei 115, Taiwan

Contents:

Supplementary Tables 1

Supplementary Figures 1-10

**Supplementary** **Table 1.** List of the interested proteins identified from fetal bovine serum by LC/MS/MS.

| Protein | Accession # of UniProKB | Protein | Score | # of Identified Peptides |
| --- | --- | --- | --- | --- |
| Apo-AI | APOA1_BOVIN | Apolipoprotein A-I | 8667 | 39 |
| A | A0A140T897 | Serum albumin | 34725 | 90 |
| B | P34955 | α1-antitrypsin | 21917 | 43 |
| C | G3X6N3 | Serotransferrin | 50178 | 96 |
| D | A0A140T897  P34955 | Serum albumin  α1-antitrypsin | 7900  3247 | 54  23 |
| D1  D2 | A0A140T897  P34955 | Serum albumin  α1-antitrypsin | 29992  8400 | 92  29 |

The MS raw data were searched against the UniProtKB BOVINE database.


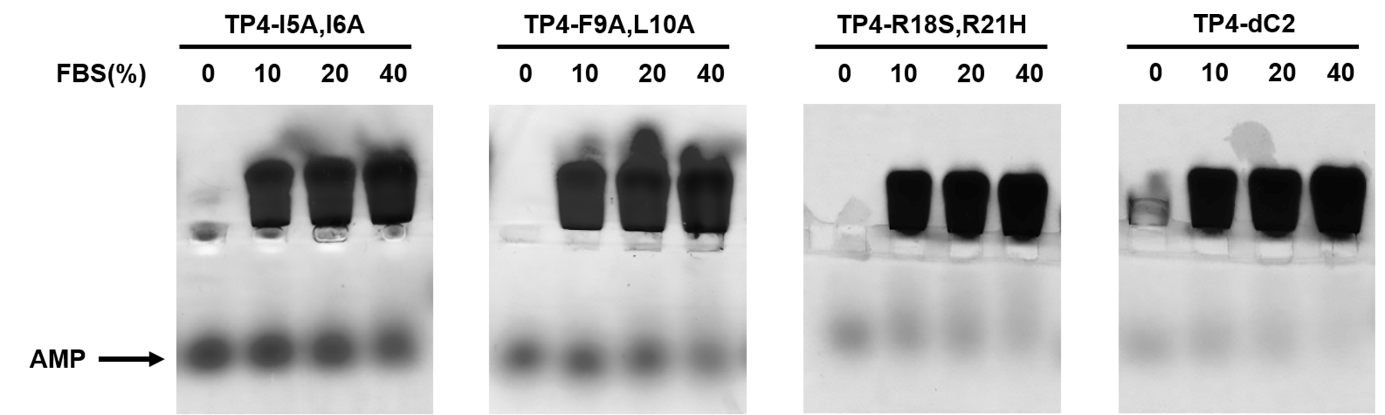


**Supplementary Figure 1.** Effect of fetal bovine serum on the band shift of TP4 mutants. Various AMPs (4μg each) were incubated with fetal bovine serum at room temperature in 10μl PC buffer for 30 min and subjected to horizontal native 8% PAGE, pH8.0 and Coomassie Blue staining.


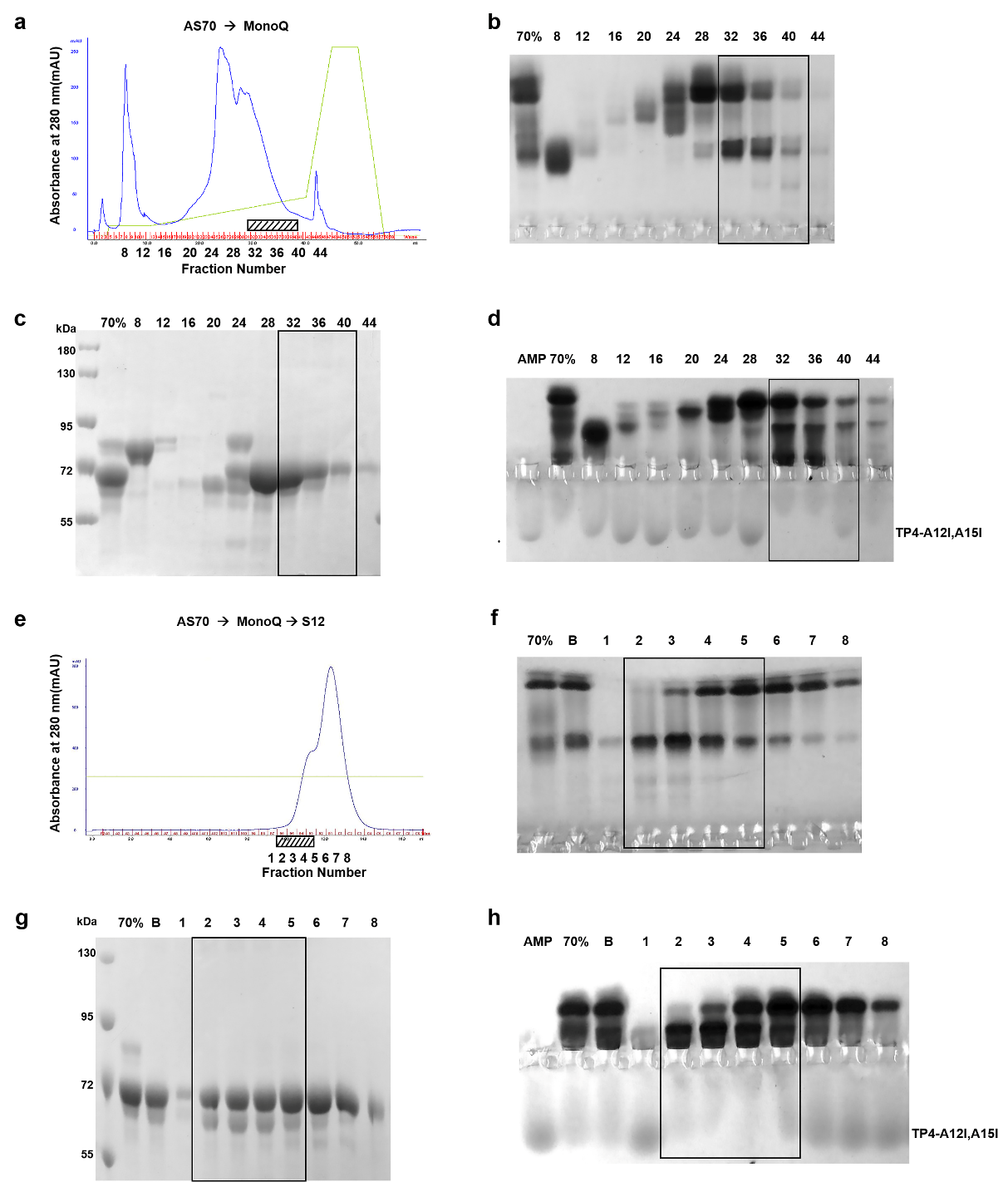


**Supplementary** **Figure 2.** Purification of serum components by fast protein liquid chromatography (FPLC). (**a**) FPLC Mono Q chromatography for the purification of serum proteins from 70%-saturated ammonium sulfate fractionation. (**b-c**) Protein composition of Mono Q column eluates analyzed by 8% horizontal native PAGE (**b**) and vertical 8% reduced SDS-PAGE (**c**). (**d**) Band shift of TP4-A12I,A15I by Mono Q column eluates. (**e**) FPLC S12 gel filtration chromatography for the purification of protein D from Mono Q column eluates. (**f-g**) Protein composition of S12 column eluates analyzed by 8% horizontal native PAGE (**f**) and vertical 8% reduced SDS-PAGE (**g**). (**h**) Band shift of TP4-A12I,A15I by S12 column eluates.

*Active fractions for TP4-binding are shown in hatch or box.


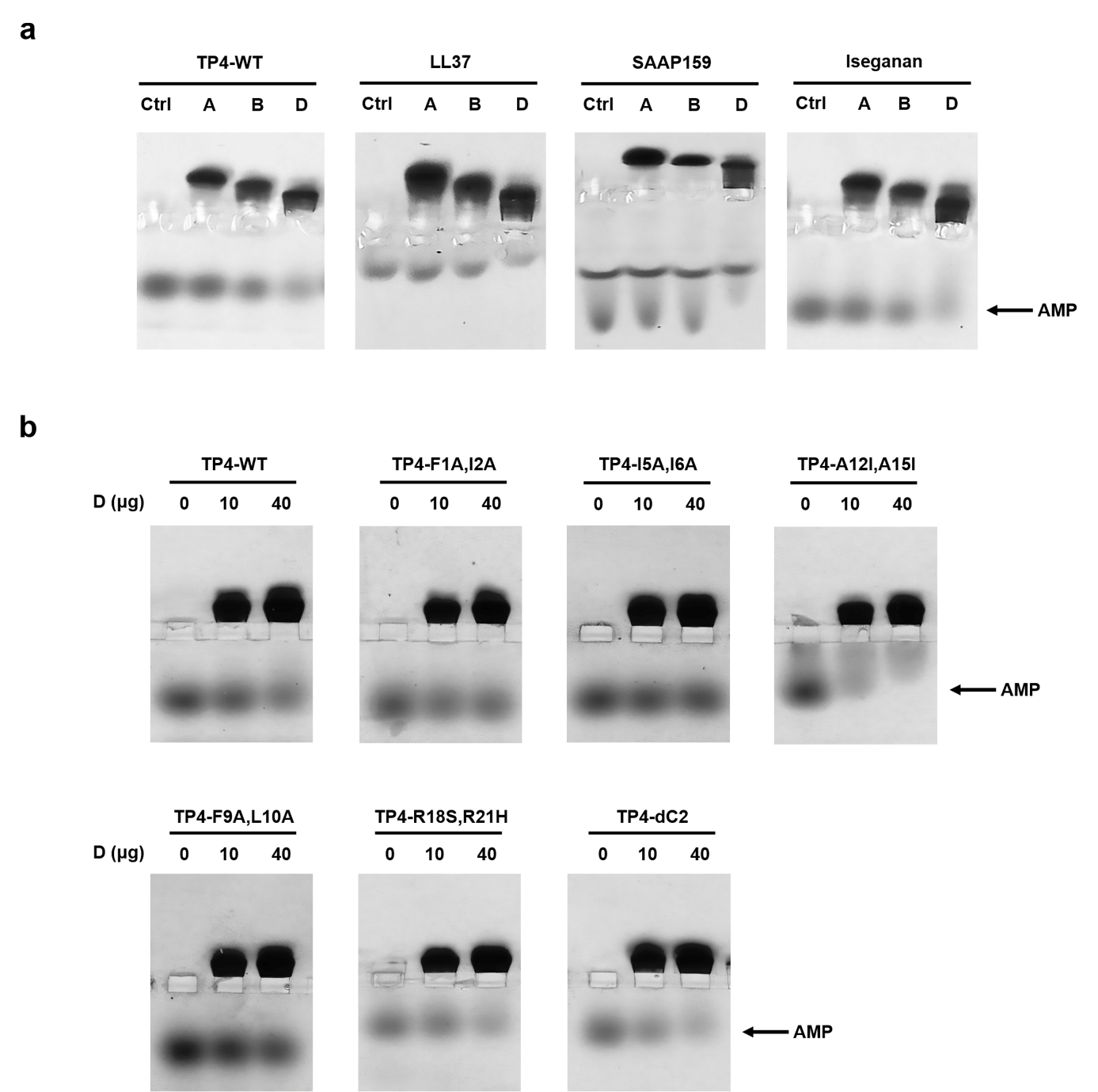


**Supplementary** **Figure 3**. Binding of purified proteins to AMPs. (**a**) Band shift of various AMPs (4μg each) by proteins A, B and D (10μg each) was analyzed by 8% horizontal native PAGE and Coomassie blue staining. (**b**) Band shift of TP4 mutants (4μg each) by proteins D was analyzed by 8% horizontal native PAGE and Coomassie blue staining.


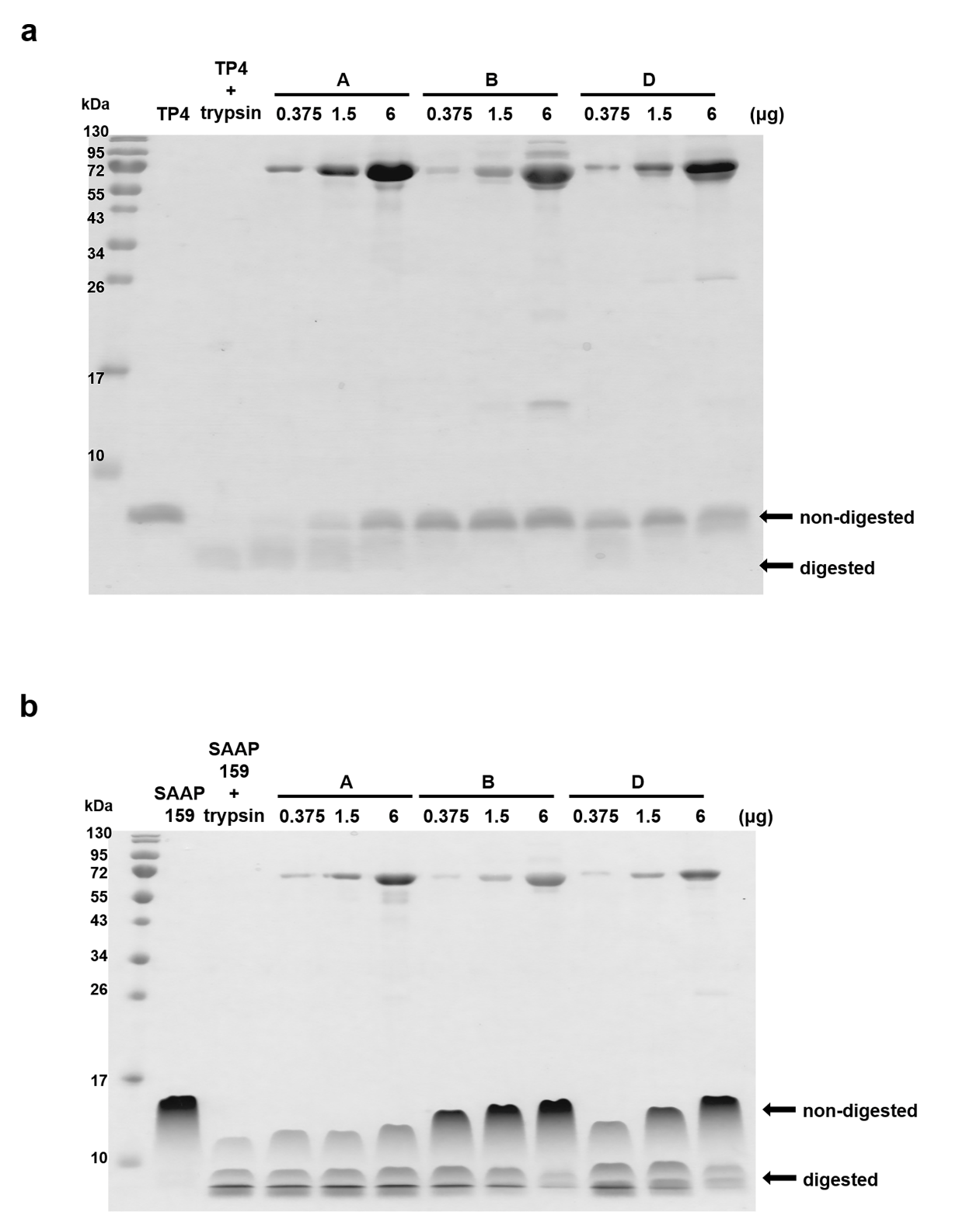


**Supplementary** **Figure 4.** Inhibition of the proteolytic activity of trypsin by proteins A, B and D. Trypsin (2ng; 0.2μg/ml) was pre-incubated with protein A, B or D first, then mixed with the substrate, TP4 (2μg; 0.2mg/ml) (**a**) or SAAP159 (4μg/; 0.4mg/ml) (**b**), in 10μl at 37°C for 30 min and analyzed by 15% reduced SDS-PAGE and Coomassie blue staining.


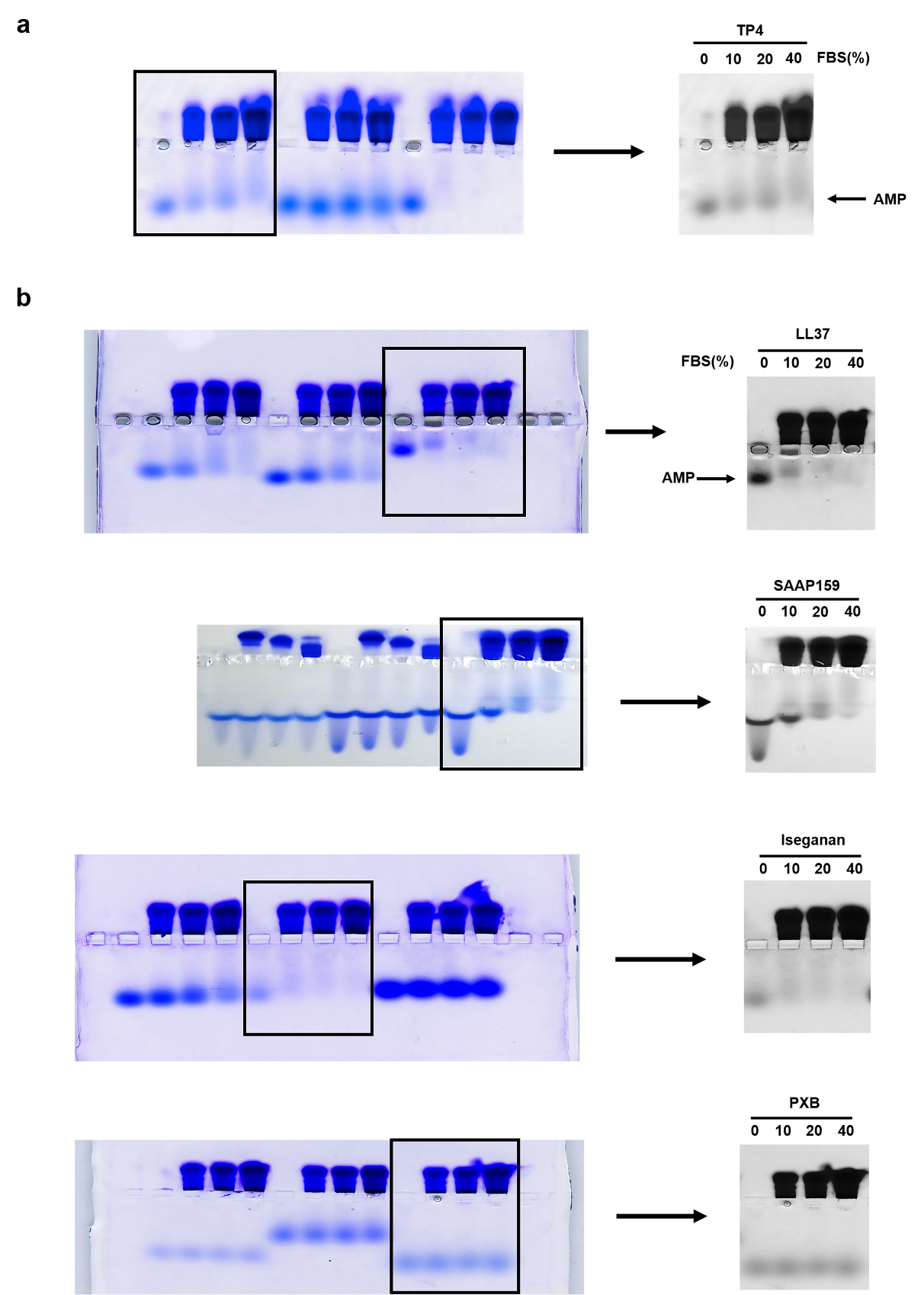


**Supplementary** **Figure 5**. Demonstration of original (left) and final (right) gels which were used in Figure 1b (**a**) and Figure 1c (**b**).


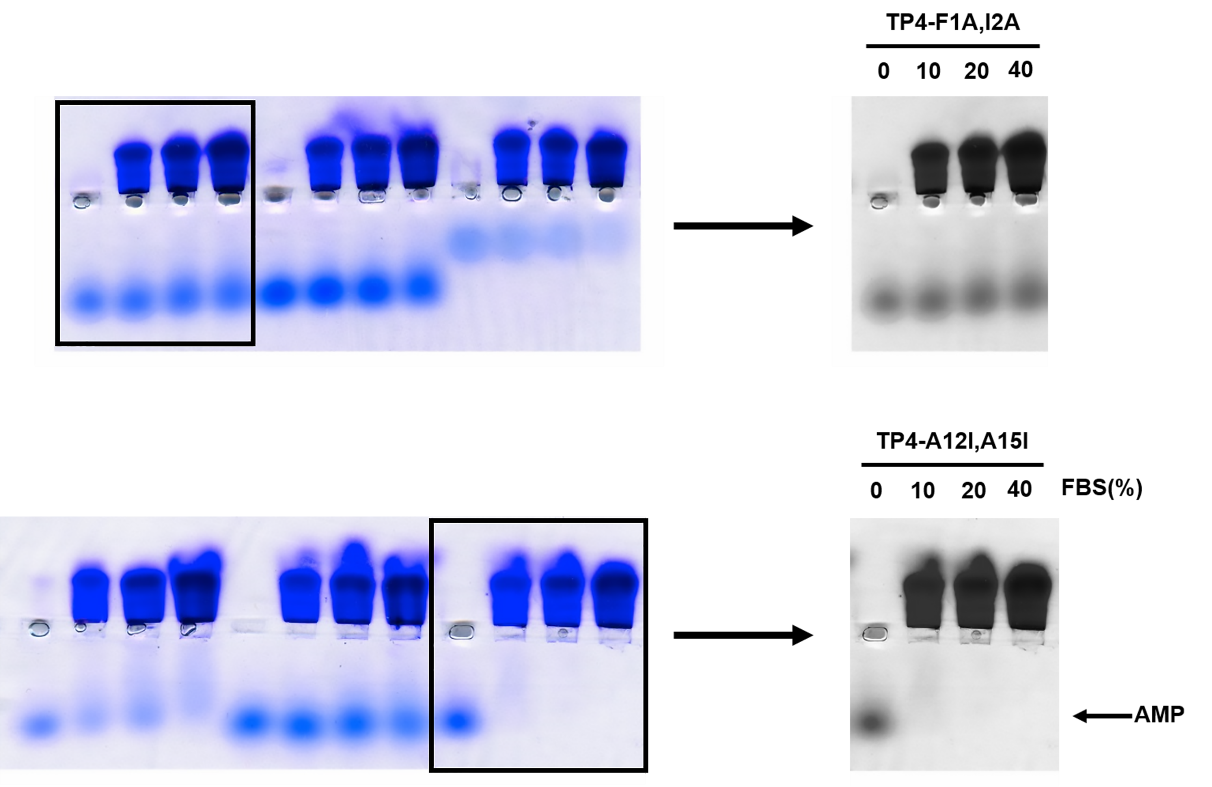


**Supplementary** **Figure 6**. Demonstration of original (left) and final (right) gels which were used in Figure 1d.


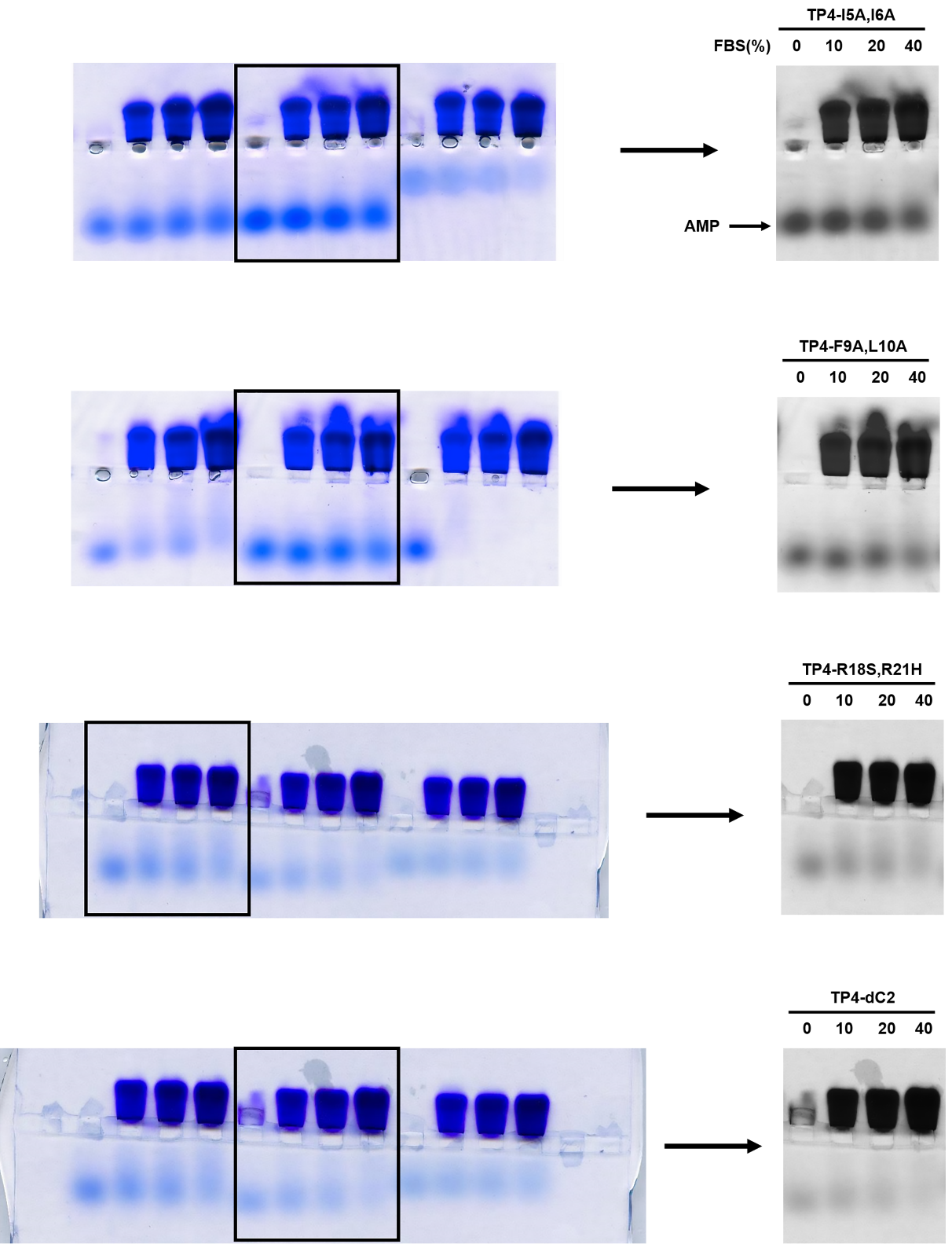


**Supplementary** **Figure 7**. Demonstration of original (left) and final (right) gels which were used in Supplementary Figure 1.


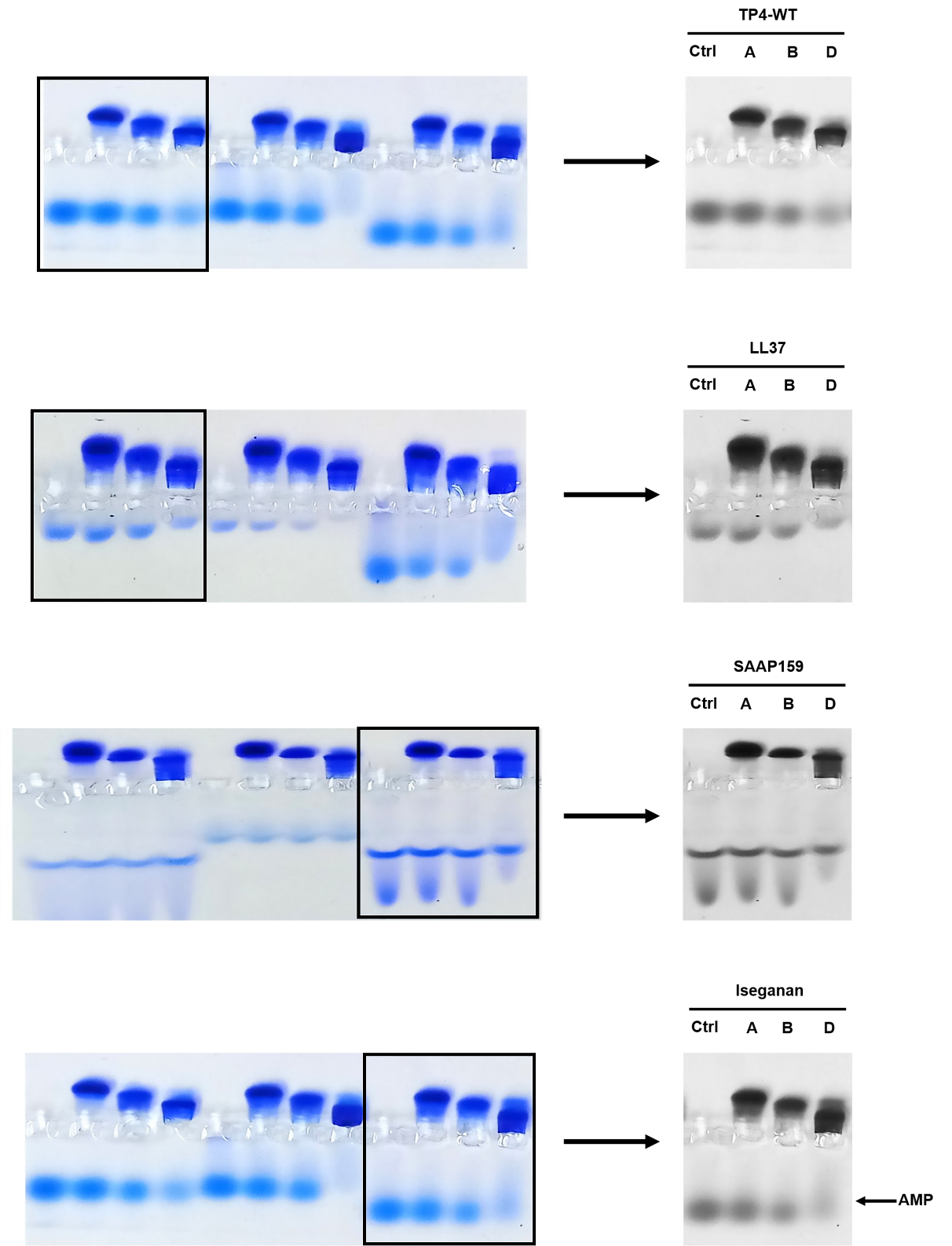


**Supplementary** **Figure 8**. Demonstration of original (left) and final (right) gels which were used in Supplementary Figure 3a.


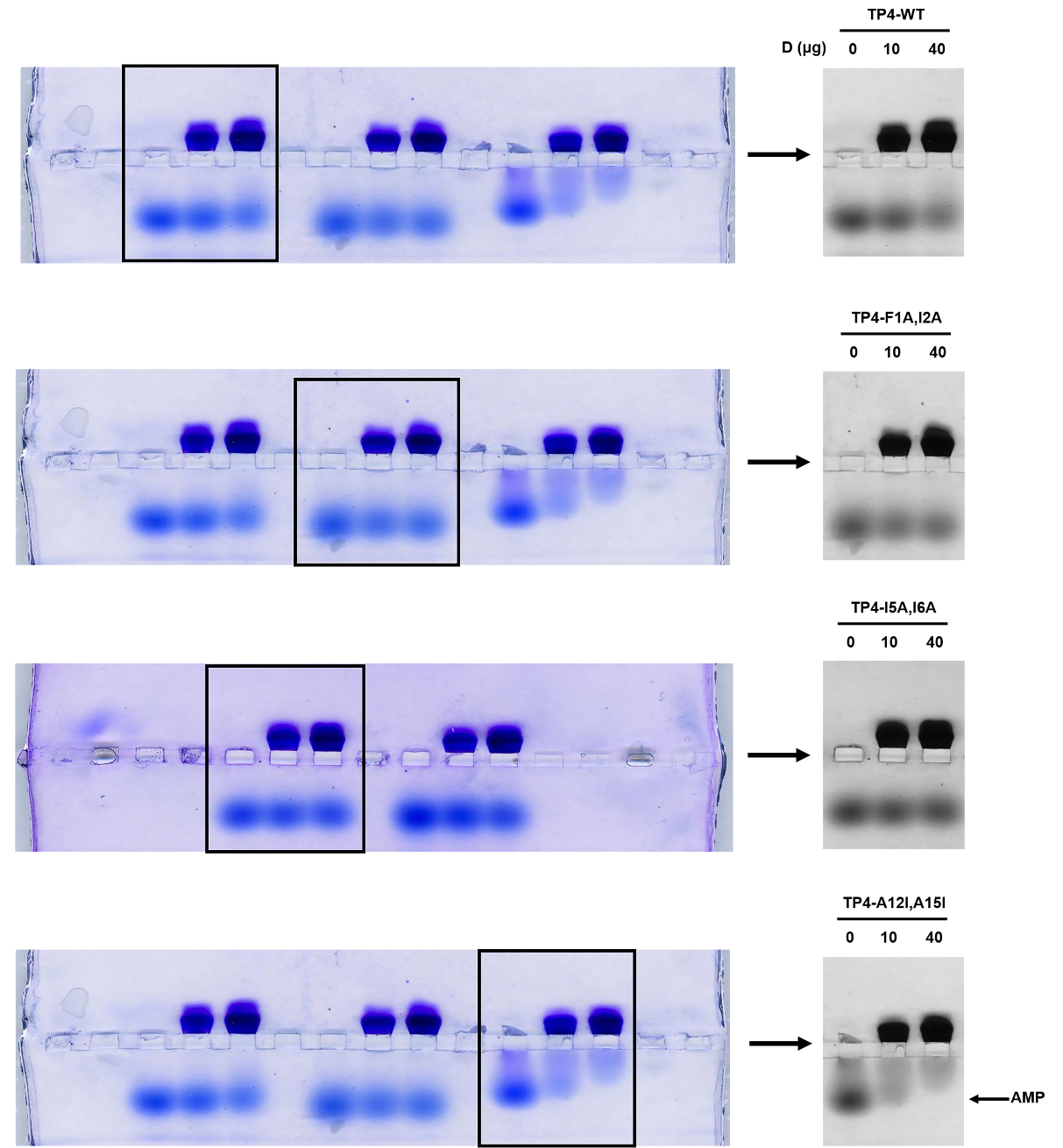


**Supplementary** **Figure 9**. Demonstration of original (left) and final (right) gels which were used in Supplementary Figure 3b.


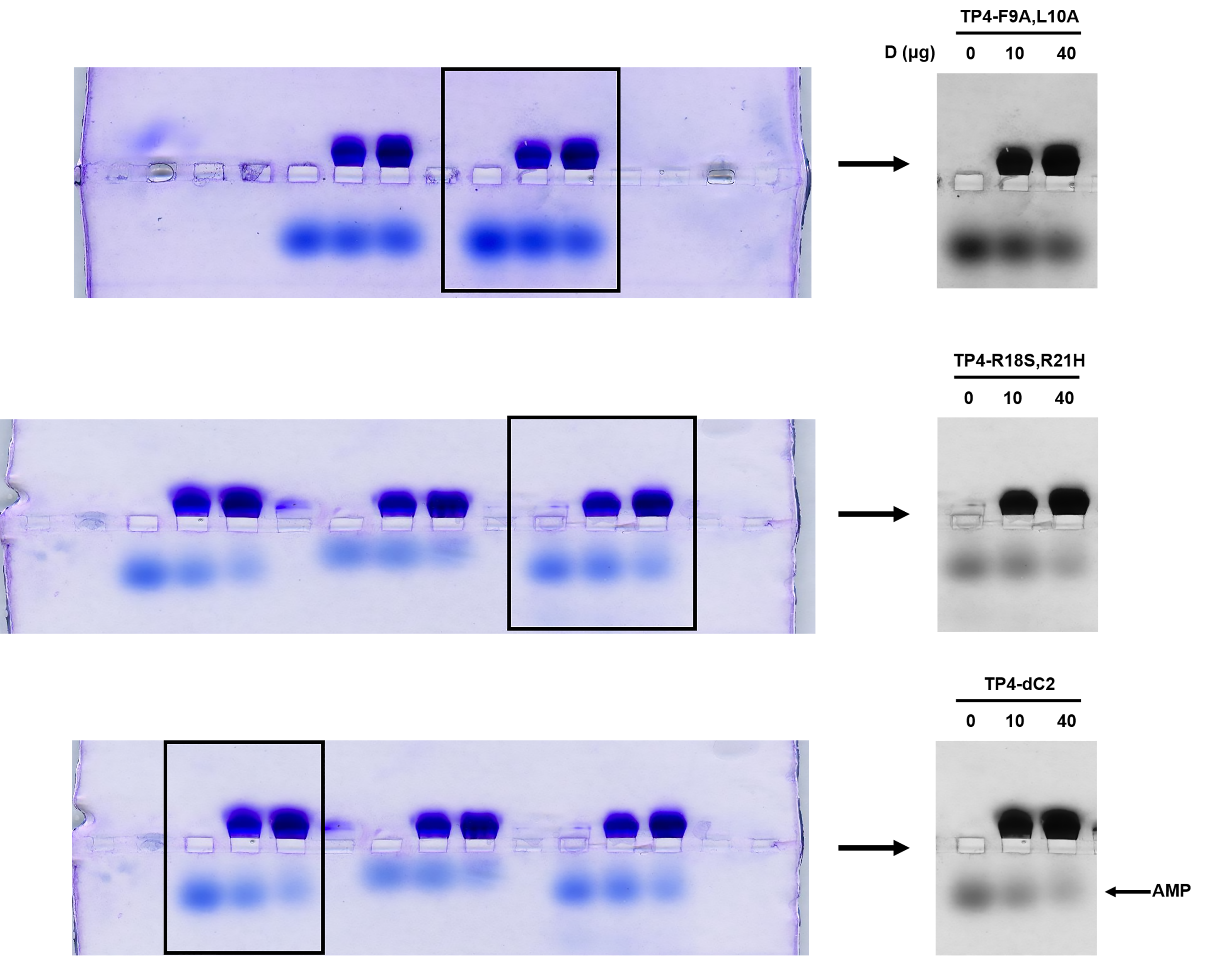


**Supplementary** **Figure 10**. Demonstration of original (left) and final (right) gels which were used in Supplementary Figure 3b.
